# Transient surface photovoltage studies of bare and Ni-filled porous silicon performed in different ambients

**DOI:** 10.1186/1556-276X-9-423

**Published:** 2014-08-21

**Authors:** Petra Granitzer, Klemens Rumpf, Yuri Strzhemechny, Puskar Chapagain

**Affiliations:** 1 Institute of Physics, Karl Franzens University Graz, Universitaetsplatz 5, Graz, 8010, Austria; 2 Department of Physics and Astronomy, Texas Christian University, 76129 Fort Worth, TX, USA

**Keywords:** Porous silicon, Electrodeposition, Surface photovoltage, 81.05.Rm, 73.20.-r, 75.50.-y, 82.45.Yz

## Abstract

Mesoporous silicon and porous silicon/Ni nanocomposites have been investigated in this work employing light-dark surface photovoltage (SPV) transients to monitor the response of surface charge dynamics to illumination changes. The samples were prepared by anodization of a highly *n*-doped silicon wafer and a subsequent electrodepositing of Ni into the pores. The resulting pores were oriented towards the surface with an average pore diameter of 60 nm and the thickness of the porous layer of approximately 40 μm. SPV was performed on a bare porous silicon as well as on a Ni-filled porous silicon in vacuum and in different gaseous environments (O_2_, N_2_, Ar). A significant difference was observed between the ‘light-on’ and ‘light-off’ SPV transients obtained in vacuum and those observed in gaseous ambiences. Such behavior could be explained by the contribution to the charge exchange in gas environments from chemisorbed and physisorbed species at the semiconductor surface.

**PACS:**

81.05.Rm; 73.20.-r; 75.50.-y; 82.45.Yz

## Background

Porous silicon (PS) exhibits numerous properties directly related to its microstructure, which in turn can be modified within a broad range of morphologies. Freshly etched PS offers a hydrogen-terminated surface. Due to the high surface area and the high reactivity, such as-etched PS oxidizes easily. It can be oxidized, e.g., by storing in air (native oxide layer) and via thermal or chemical treatment. Oxidation is the main aging aspect and therefore, knowledge about the oxidation state of the surface is of importance. Light illumination decreases the H-termination of as-etched samples. Photoirradiation in an oxygen ambient causes photo-oxidation at the surface and thus accelerates aging of the material. Chemical stability of PS is one of the preconditions rendering the material compatible with any application for which surface modification is desirable.

For practical applications of PS in solar cells, light-emitting diodes, chemical and gas sensors, etc., it is thus desirable to understand the behavior of PS in different ambients. The surface of PS is known to be sensitive to the surrounding environments [1-3]. For example, surface electronic states could be affected by gas species by physisorption, chemisorption, or desorption from the surface [4,5]. On the other hand, filling of PS with magnetic metals [6,7] is of interest due to both the distinct properties of the nanosized deposits and the employment of silicon as the base material, key for integration in microtechnology.

In this work, we employed transient surface photovoltage (SPV) to monitor the response of the surface electronic structure of PS to the change of ambience. SPV probes light-induced variations in the electric potential of a studied surface, mostly in semiconductors and insulators [8]. Surface potential barrier in semiconductors is formed due to charges trapped in surface states. The illumination-induced changes of the surface barrier depend strongly on the surface/subsurface electronic structure, which, in turn, can be affected by the physisorbed and chemisorbed species. In transient SPV experiments, the surface potential is monitored as a function of illumination time which can provide information about the different transport mechanisms in semiconductors. SPV is a non-destructive and a highly surface-sensitive tool, which can be operated in different environments. A number of SPV studies on PS were reported in the literature, with most of them performed in ambient air [9-11]. Some authors addressed the influence of the surface chemistry on the SPV response in PS, revealing dependence on the microstructure and chemical environment of the surface [12-14]. However, there was insufficient experimental evidence of the influence of the surface environment (such as vacuum vs. gas) on the SPV response in PS. To address this, in our work, bare PS specimens as well as samples with embedded Ni deposits have been measured by SPV in vacuum and in different gaseous environments (O_2_, N_2_, Ar). It was revealed that the illumination-induced charge transport mechanisms were strongly influenced by the experimental ambiences. The behavior of the SPV transients obtained for gaseous environment was significantly different from that observed in high vacuum.

## Methods

**Figure 1:**
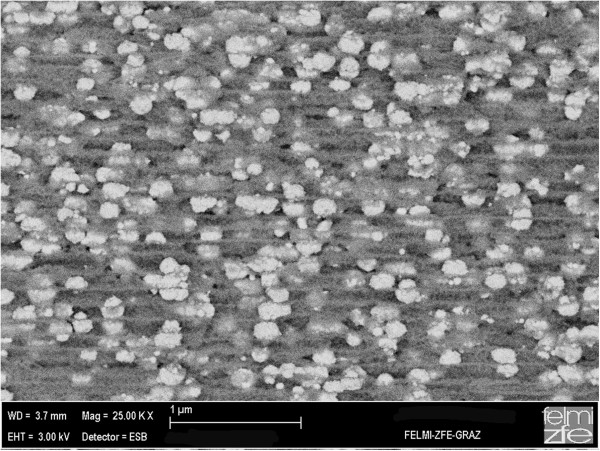
**SEM image of a Ni-filled PS sample.** SEM image (formed by back-scattered electrons) of a Ni-filled PS sample with a high density of Ni-particles in the pores with an average size of 100 nm.

The investigated PS samples were fabricated by anodization in aqueous hydrofluoric acid solution. Highly *n*-doped silicon was used as a substrate. The produced morphology revealed average pore diameters of 60 nm and a thickness of the porous layer of about 40 μm as determined by the scanning electron microscopy (SEM). Ni-nanostructures were electrochemically deposited within the pores of these templates. The size of the Ni-deposits ranged between 60 and 200 nm, whereas their diameter always correlated with the pore diameter. Figure 1 shows an SEM image of a Ni-filled PS sample with deposits of approximately 100 nm in size. Details of the fabrication process of the PS/Ni nanocomposite can be found in an earlier publication [15]. The light-dark transient SPV was employed using a broad-spectrum incident white light, which included super-bandgap wavelengths. The surface was first allowed to saturate in light, and then to reach equilibrium in the dark. SPV signal was monitored using the Kelvin probe method, a non-contact technique utilized to measure contact potential difference (CPD) between the sample surface and the probe [8]. Characterization of a bare PS and a Ni-filled PS using SPV transients for different environments were performed in high vacuum as well as in O_2_, N_2_ and Ar.

## Results and discussion

**Figure 2:**
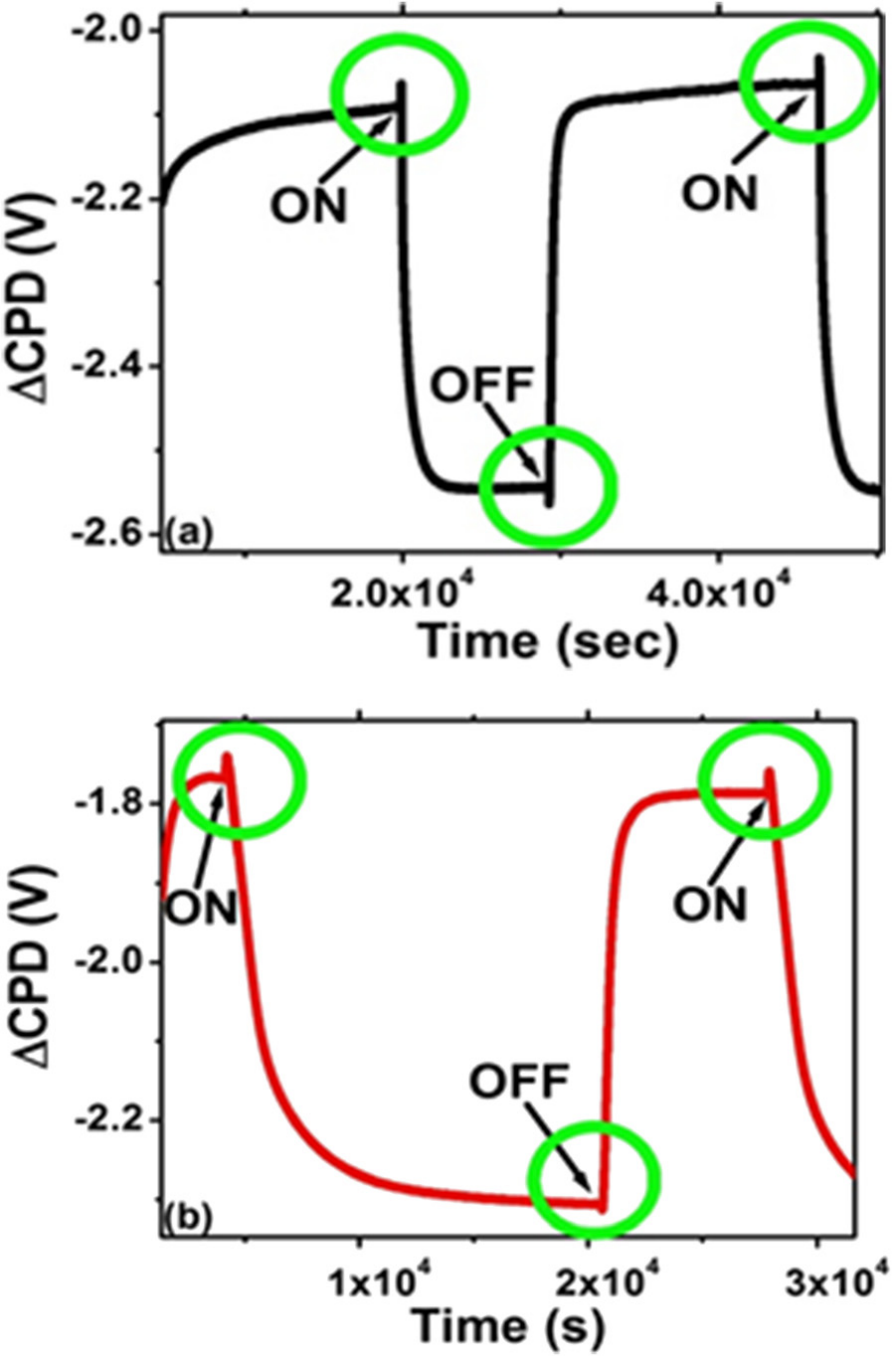
**SPV transients in gaseous environments. (a)** Bare PS in N_2_. **(b)** Ni-filled PS in O_2_.

**Figure 3:**
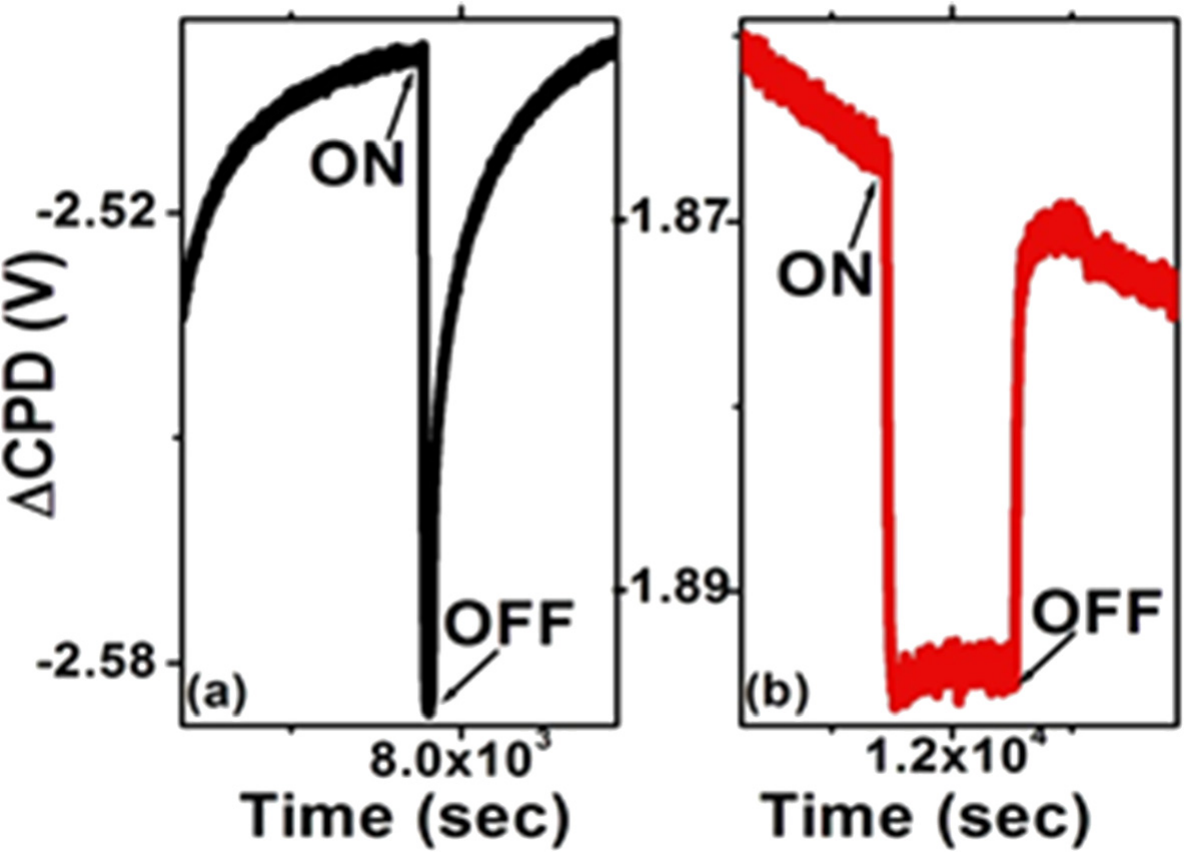
**SPV transients in vacuum. (a)** Bare PS. **(b)** Ni-filled PS.

**Figure 4:**
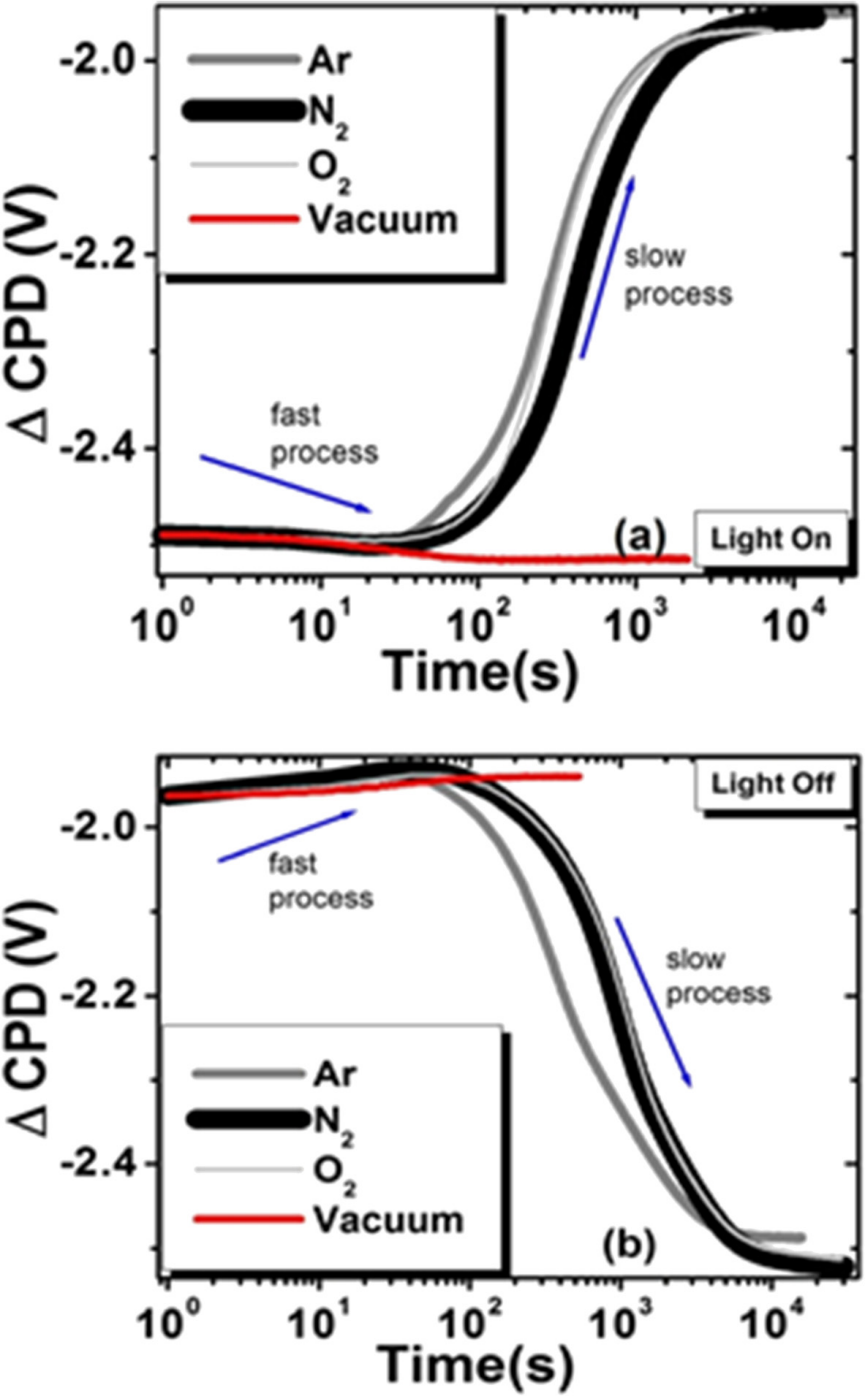
**SPV transients in different gas environments for Ni-filled PS on a logarithmic time scale. (a)** ‘Light-on’ transient. **(b)** ‘Light-off’ transient.

SPV transients for both types of samples in different gases show anomalous spikes of SPV during both ‘light-on’ and ‘light-off’ events (Figure 2). Similar behavior is observed for all three gaseous environments. After obtaining the SPV transients in these gas ambients, the experimental chamber was evacuated and then the SPV transients were obtained in vacuum.As a result, we observed that the PS surface was very sensitive to the experimental ambient, as one can see from Figure 3. In vacuum, the sharp SPV spikes disappeared whereas the light-on and light-off saturation times became dissimilar. Resolving the SPV transients obtained in gaseous environments on the logarithmic time scale (cf. Figure 4), one can see that these curves contain both fast and slow components with opposite contributions to charge dynamics. The initial fast process in the case of light-on and light-off events in the gaseous environments occurs over a time scale of tens of seconds, whereas the entire event until saturation is in the range of thousands of seconds. However, the transients observed in vacuum revealed only one relatively fast process. Since the fast process is always present regardless of the ambient conditions, we believe that it is related to the charge recombination occurring in PS. On the other hand, the slow process is present only in the gaseous environments suggesting that it might be related to the non-vacuum ambient.

A detailed discussion of fast and slow SPV transients can be found in ref. [9]. Coexisting slow and fast charge transfer processes were reported for wide-bandgap materials and analyzed theoretically by Reschikov et al. in [16-18], where it was proposed that the fast and slow components arise due to both internal contributions (related to surface/interface states) and external phenomena (related to the ambient environment). We suggest that such model could be applicable here considering a thin native oxide layer on the silicon surface. It is likely that physisorption, chemisorption, or desorption of gas species govern the observed charge dynamics. In a gaseous environment, both the internal and external charge transfer mechanisms occur in PS simultaneously but on different time scales resulting in non-trivial transients. The initial fast process during the light-on transient could be related to the drift of the illumination-induced electrons in Si towards the bulk and holes towards the Si/oxide interface due to the electric field in the space charge region (cf. [8]). On the other hand, the electrons in the trap states at the interface may recombine with the flux of holes from the Si side leading to the initial decrease of the CPD in the light-on transient. The decrease in the band bending reduces the depletion width and the barrier height. More electrons can now cross the barrier, tunnel through the oxide layer and become captured by the physisorbed gas species at the free surface and convert them into chemisorbed ones. This increases the negative charge at the free surface and consequently the band bending yielding a slow increase in the CPD of the light-on transient. However, when similar measurements were performed in vacuum, slow components were absent in transients (Figure 3). We propose that in vacuum, the physisorbed species could be removed from the surface during evacuation. Thus, only the PS internal mechanism would contribute to the SPV transients in vacuum during the light-on process, explaining the observed behavior.

In addition, our experiments show that there is no difference between the SPV transients for the bare and Ni-filled PS. This fact indicates that the metal deposits inside the pores do not influence the optoelectronic transport properties of PS, an important observation considering potential multifunctional (magnetic/chemical/pressure) sensor applications of Ni-filled PS.

## Conclusions

In this work, employing transient SPV, we studied charge dynamics of mesoporous silicon and Ni-filled mesoporous silicon in different gas ambients and vacuum. We found that SPV transients for both types of samples in gaseous environments showed a non-trivial behavior during the light-on and light-off events. Vacuum transients showed a different behavior without the slow component. The time scale of the light-on and light-off events in vacuum and in gaseous ambients differs by three orders of magnitude. We suggest that the observed behavior is related to the charge exchange between the silicon/oxide interface and free-surface adsorbates.

## Competing interests

The authors declare that they have no competing interests.

## Authors’ contributions

PG and KR fabricated the porous silicon and Ni-filled porous silicon samples, and PC and YS performed the surface photovoltage transient measurements. All authors discussed the data and prepared the manuscript. All authors read and approved the final manuscript.
